# Urinary Immunoglobulin G to Albumin Ratio and N-Acetyl-Beta-D-Glucosaminidase as Early Predictors of Therapeutic Response in ANCA-Associated Glomerulonephritis

**DOI:** 10.1371/journal.pone.0081703

**Published:** 2013-12-13

**Authors:** Marija Mravljak, Alenka Vizjak, Dusan Ferluga, Jernej Pajek, Damjan Kovac, Andrej Skoberne, Andreja Ales Rigler, Radoslav Kveder, Andrej Kosir, Jelka Lindic

**Affiliations:** 1 Department of Nephrology, University Medical Center Ljubljana, Ljubljana, Slovenia; 2 Institute of Pathology, Faculty of Medicine, University of Ljubljana, Ljubljana, Slovenia; 3 Faculty of Electrical Engineering, University of Ljubljana, Ljubljana, Slovenia; University of Texas Health Science Center at Houston, United States of America

## Abstract

**Background:**

The aim of our study was to evaluate the prognostic value of glomerular and tubular proteinuria and tubular enzymuria as early indicators of therapeutic response to induction therapy with i.v. pulse cyclophosphamide (CyC) and methylprednisolone (MP) in patients with antineutrophil cytoplasmic antibody (ANCA) associated glomerulonephritis.

**Methods and Findings:**

An observational single-center study was conducted in 30 patients with ANCA-associated glomerulonephritis. Patients were divided into subgroups with good or poor response to CyC therapy according to clinical and laboratory parameters. The diagnosis of ANCA-associated glomerulonephritis was based on the Chapel-Hill disease definitions. Good response to induction therapy was significantly associated with higher absolute values of urine N-acetyl-beta-D-glucosaminidase (NAG) to creatinine ratio (above 14.83 microcat/mol) and urine immunoglobulin G (IgG) to albumin ratio (above 0.09) at the time of diagnosis, while albuminuria or proteinuria did not have any early predictive value. The remission of renal disease was anticipated as early as 3 months after introduction of induction therapy in patients with reduction of urine NAG to creatinine ratio below the baseline value and in patients with at least 24% rise in eGFR.

**Conclusions:**

Urine IgG to albumin and urine NAG to creatinine ratio are better early predictors of treatment response in patients with ANCA-associated glomerulonephritis than proteinuria or albuminuria.

## Introduction

Antineutrophil cytoplasmic antibody (ANCA)-associated vasculitides are chronic multisystemic autoimmune diseases with a prevalence of 2.5/100,000 that follow an unpredictable course [1,2]. Despite improved diagnostic procedures and efficient induction and maintenance immunosuppressive therapy, 11–57% of patients have a relapsing disease that results in high cumulative dose of potentially toxic cyclophosphamide (CyC) [3-8]. Therefore it is important to detect the optimal response to induction therapy as soon as possible to be able to proceed with less toxic immunosuppressive maintenance therapy or, in patients with suboptimal response to immunosuppressive therapy, to intensify the induction therapy in a timely manner. The gold standard for diagnosis of ANCA-associated glomerulonephritis is kidney biopsy, but it is less suitable for evaluation of therapeutic response to induction therapy due to possible complications of invasive procedure [9–13]. 

Urinary protein excretion is a cardinal sign of kidney disease and a strong predictor of its progression [14]. When proteinuria is detected in patients with glomerulonephritis, it is a consequence of active inflammation and/or the result of chronic injury. Glomerular damage is characterized by glomerular proteinuria, which is measured as albuminuria, a widely recognized predictor of renal disease progression, increased morbidity and mortality, and urine excretion of IgG or IgM, while tubular damage is characterized by urinary excretion of alpha-1-microglobulin and N-acetyl-beta-D-glucosaminidase (NAG) [15-22]. In ANCA-associated glomerulonephritis, urinary IgM excretion has been studied and was considered as a predictor of poor disease outcome [23]. In some primary glomerulonephritides with nephrotic syndrome, increased urinary levels of NAG and alpha-1-microglobulin were helpful to predict the treatment outcome [24-26], but less is known about differential prognostic value of tubular proteinuria or enzymuria in ANCA-associated glomerulonephritis.

Our retrospective study was conducted to evaluate the prognostic significance of glomerular and tubular proteinuria and enzymuria as possible early indicators of therapeutic response to induction therapy with i.v. pulse CyC and steroids in patients with ANCA-associated glomerulonephritis.

## Subjects and Methods

All data were retrieved retrospectively at the Department of Nephrology, University Medical Centre Ljubljana, Slovenia, while systemically reviewing medical records on patients’ history, laboratory results and medication at the time of diagnosis and during follow-up. The study was approved by the National Medical Ethics Committee of the Republic of Slovenia (permit number 109/09/10). The data were analysed anonymously. Clinical investigation was conducted according to the principles expressed in the Declaration of Helsinki. The National Medical Ethics Committee that approved the protocol of the study did not require explicit and specific patients' consent to the use of their anonymized medical data. The duty of obtaining the patients' consent for using their data has been waived in accordance with the Article 44 of the Slovenian Patient's Rights Act, according to which anonymized medical data may be reported in research papers when patient's identity cannot be recovered. This certainly applies to research studies where the published results only contain aggregate data from patient groups, as indeed is the case in the study concerned.

### Patients

Patients were included if newly diagnosed with renal vasculitis between 1 January 2005 and 31 March 2011. The diagnosis of ANCA-associated vasculitis was based on the Chapel-Hill disease definitions and included microscopic poliangiitis, granulomatous poliangiitis, and renal-limited pauci-immune necrotizing crescentic glomerulonephritis [1,27-29]. 

Criteria for exclusion were the coexistence of anti-glomerular basement membrane disease or other autoimmune disease, hepatitis B antigenemia, hepatitis C or human immunodeficiency virus infection, drug induced glomerulonephritis, malignancy, the use of immunosuppressive therapy within the previous year, pregnancy and an age of less than 18 years.

### Assesment of disease activity and treatment

Renal involvement was defined by the presence of proteinuria, erythrocyturia with or without red blood cell casts, decreased calculated estimated glomerular filtration rate (eGFR) according to the Modification of Diet in Renal Disease (MDRD) equation, elevated titer of antineutrophil cytoplasmic antibody (ANCA), and typical histological features of the disease obtained by representative percutaneous renal biopsy specimens, when possible.

All patients were treated according to local treatment guidelines with initial induction therapy: i.v. pulse of CyC 750 mg every 3 to 4 weeks (or 500 mg if ≥70 years old or eGFR >20 ml/min/1.73 m^2^ or body weight <60 kg) and methylprednisolone (MP) i.v. pulse (250-500 mg/day) for 3 consecutive days, followed by oral dose of 0.8 mg/kg body weight (or 0.4 mg/kg body weight if ≥70 years old or if eGFR < 20 ml/min/1.73 m^2^ or body weight <60 kg), tapered after 4 weeks gradually to maintenance dose of 0.08 mg/kg body weight. Induction therapy with i.v. CyC was continued for 6 consecutive applications. In patients with inadequate renal response, we consecutively increased the dose of i.v. pulse CyC. Maintenance therapy consisted of azathioprin and MP, in case of intolerance to azathioprine, mycophenolate mophetil (MMF) was introduced. If patients with pulmonary involvement were dialysis dependent after 3 months of induction therapy, the maintenance therapy consisted of MP thereafter. In case of renal-limited disease and dialysis dependency, the induction therapy was stopped completely after 3 months. 

We calculated the Birmingham Vasculitis Activity Score (BVAS) version 3 every month [27]. All patients were treated with proton pump inhibitors, active vitamin D3 as osteoporosis prevention measures, and prophylactic dose of trimethoprim-sulfamethoxazole. 

Remission was defined as the absence of clinical or laboratory evidence of disease activity according to BVAS version 3 (BVAS zero or BVAS ≤5 if all scores were due to persistent haematuria or proteinuria in the presence of stable or improving renal function as measured by eGFR) [27]. Refractory disease was defined as failure to attain remission following induction therapy according to local treatment guidelines. Relapse was defined as the re-occurrence or new onset of disease activity attributable to active inflammation after achieving remission [28–30]. 

Renal response was evaluated after 6 months of induction therapy and was defined in patients with rapidly progressive glomerulonephritis as 30% improvement in eGFR or dialysis independence if they have been dialysis dependent at the time of diagnosis, or as stabilization or improvement of renal function in patients with chronic nephritic syndrome, with concomitant reduction of erythrocyturia (<10 red blood cells/high power field) and proteinuria (≤0.6 g/day) irrespective of clinical presentation of the renal disease. Patients fulfilling the criteria for renal response were designated as responders to induction therapy, all others were designated as non-responders. 

### Laboratory analysis

Blood samples and urine specimens were obtained on the first days after each admission. Glomerular filtration rate was estimated by using the MDRD study equation (eGFR) [31]. Morning urine protein to creatinine ratio was measured as a surrogate marker of 24-hour urine protein excretion. Glomerular proteinuria was measured as urine albumin to creatinine ratio and IgG to creatinine ratio. Tubular enzymuria and proteinuria were measured as NAG to creatinine and α-1-microglobulin to creatinine ratio. Urine albumin, immunoglobulin G (IgG) and α-1-microglobulin were determined by immunonephelometry (BN ProSpec, Siemens, Germany). NAG was determined by spectrophotometric assay using 3-cresolsulfonphtalein-N-acetyl-beta-D-glucosaminidase, (Olympus AU400, Olympus, Japan), and serum and urine creatinine by modified Jaffe’s method (Dimension Xpand, Siemens, Germany). Enzyme-linked immunosorbent assay (ELISA) was used to analyze ANCA by indirect immunofluorescence (IF) as well as for PR3-ANCA and MPO-ANCA by IF tests, and ELISA systems used for detection were manufactured by Euroimmune, Lübeck, Germany, and Wieslab, Lund, Sweden. Renal biopsies were examined by standard light and immunofluorescence microscopy techniques. Histopathologic findings were assessed and were divided into four categories, named focal, crescentic, sclerotic and mixed (10), and the number of normal glomeruli was reported as well [11].

### Statistical analysis

Statistical analysis was performed with SPSS statistical software (version 18). Medians and ranges are reported for non-normally distributed data and means + standard deviation (SD) are reported for normally distributed data. 

The demographic characteristics of responders and non-responders were compared; categorical variables were analyzed by the Fisher’s exact test. The differences between the two groups were compared by the Mann-Whitney U-test, and in each group with the Sign test. P-values of less than 0.05 were considered to indicate statistical significance. Receiver operating characteristic (ROC) curve with area under the curve (AUC) was performed as a plot of test sensitivity and specificity and cutoff value was determined as the maximum sum of sensitivity and specificity.

## Results

A total of 30 patients with ANCA-associated glomerulonephritis were included in the study and renal response was evaluated after 6 months of induction therapy. Demographic and clinical characteristics of all patients, responders and non-responders, are shown in Table 1. All were European Caucasians from a single center. The mean time of clinically active disease before initiation of induction therapy was 8.9 + 4 months (range 0.5–48.0 months) and observation treatment period 31 + 12 months (range 23–38 months), without any difference between responders and non-responders. The use of antiproteinuric therapy – angiotensin-converting enzyme inhibitors (ACE inhibitors) and/or angiotesin II receptor antagonists – was not statistically significant between both groups: 18 responders (82%) versus 6 non-responders (75%).

**Table 1:** Demographic and clinical characteristics of patients with ANCA-associated glomerulonephritis in all patients, in responders and in non-responders at the time of diagnosis.

Category	Subcategory	All patients (N=30)	Responders (N=22)	Non-responders (N=8)	p
Age (year)	Mean ± SD	63.6 ± 12.7	62.4 ± 13.3	66.8 ± 11.1	0.22
	Range	31–88	31–77	54–88	
Sex (No.)	Women	18	12	6	0.41
	Men	12	10	2	0.28
Diagnosis (No.)	MPO-ANCA systemic vasculitis	16	11	5	0.45
	PR3-ANCA systemic vasculitis	8	7	1	0.37
	Renal limited ANCA glomerulonephritis	6	4	2	0.57
Other affected organs (No.)	General symptoms	27	20	7	0.51
	Skin	6	4	2	0.65
	Eyes/mucous membranes	6	5	1	0.76
	Ear/nose/throat	9	8	1	0.41
	Lung	18	12	6	0.55
	Cardiovascular	3	1	2	0.82
	Nervous system	9	7	2	0.94

At the time of admission to the hospital, all patients had microscopic erythrocyturia and proteinuria. Twelve of the patients (40%) were dialysis-dependent (8 responders and 4 non-responders). Twenty-two patients had positive p-ANCA (21 patients had MPO-ANCA and one patient unspecified antigen); 6 of them were non-responders. Eight patients had positive c-ANCA (all PR3-ANCA); 2 of them were non-responders. One responder was positive for MPO-and PR3-ANCA. There was no statistical difference in ANCA presentation between both groups. After 6 months of therapy, there was no statistically significant difference in decrease of titers between responders and non-responders (PR3-ANCA 80.7% versus 87.5%, and MPO-ANCA 64% versus 54.4%). 

Renal biopsy specimens were available in 24 patients (80%), with mean of 20 + 7 glomeruli per biopsy (range 9–44 glomeruli per biopsy). On direct immunofluorescence, all histologies were pauci-immune. Pathohistological characteristics of kidney biopsy in responders and non-responders were not statistically different and as such without predictive value for renal response: percentage of normal glomeruli was 21.1% versus 16.5% (p = 0.67), global sclerosis 27.1% versus 30.4% (p = 0.52), segmental sclerosis 24.2% versus 31.1% (p = 0.24), crescents 50% versus 49% (p = 0.81) and interstitial fibrosis 71.6% versus 80% (p = 0.15). According to pathohistological classification of ANCA-associated glomerulonephritis 2 responders had crescentic class (p = 1.00), 12 responders and 5 non-responders had mixed class (p = 1.00), 3 responders and 2 non-responders had sclerotic class (p = 0.58), and no patient had focal class of ANCA-associated glomerulonephritis. 

No significant differences between responders or non-responders were observed in C-reactive protein (CRP) value at the time of diagnosis (49.7 ± 3 mg/l versus 25.0 ± 2 mg/l, p = 0.30), nor in damage extension index (DEI) at the time of diagnosis (7.2 ± 2 versus 7.9 ± 4 points, p = 0.81), or vasculitis damage index (VDI) score after 6 months of induction therapy (2.5 ±1 versus 3.7 ± 2 points). After 6 months of induction therapy, the radiological signs of pulmonary fibrosis were evident in 1 responder and 4 non-responders (p = 0.011), but urine protein to creatinine, albumin to creatinine, IgG to creatinine, IgG to albumin, NAG to creatinine and α-1-microglobulin to creatinine ratios had no predictive values for pulmonary response. 

Calculated mean and range of BVAS, eGFR, urine albumin to creatinine, urine IgG to creatinine, and urine α-1-microglobulin to creatinine at the time of diagnosis, 3 and 6 months after starting of induction therapy, are presented in Table 2. 

**Table 2:** Calculated BVAS score and eGFR at the time of diagnosis (start), 3, 6 and 12 months after induction therapy, and urine albumin to creatinine, IgG to creatinine, and α-1-microglobulin to creatinine in patients with ANCA-associated glomerulonephritis (responders and non-responders) at the time of diagnosis (start), 3 and 6 months after induction therapy with P-value to indicate statistical significance between responders and non-responders.

Category	Subcategory	Responders, mean (95% CI), (range)	Non-responders, mean (95% CI), (range)	p
BVAS	start	23.3 (19.8–26.9)	23.7 (18.1–29.2)	0.43
	3 months	4.4 (2.6–6.3)	6.3 (2.7–9.9)	0.24
	6 months	2.6 (1.7–3.5)	4.5 (2.4–6.5)	0.07
	12 months	1.9* (1.0–2.8)	4.3** (2.0–6.6)	0.03
eGFR (ml/min/1.73 m^2^)	start	20.5 (12.2–28.8)	12.0 (5.8–18.1)	0.34
	3 months	29.7 (22.8–36.5)	12.3 (4.2–20.5)	0.13
	6 months	35.9 (26.2–45.5)	15.0 (5.7–24.2)	0.006
	12 months	37.7** (27.7–47.8)	7.2* (3.4–12.0)	0.005
Albumin/creatinine (g/mol)	start	969.9 (646.9–1318.5)	842.4 (111.5–1574.3)	0.27
	3 months	341.5 (241.5–442.4)	807.0 (61.9–1548.6)	0.29
	6 months	335.3* (140.7–530.9)	1717.6 (−1535.3–4964.6)	0.03
IgG/creatinine (g/mol)	start	20.5 (7.3–33.8)	15.0 (−3.8–33.9)	0.776
	3 months	2.9 (1.9–4.0)	8.0 (−2.7–18.8)	0.098
	6 months	1.9** (0.9–2.8)	5.7 (2.4–9.0)	0.009
α-1-microglobulin/creatinine (g/mol)	start	9.8 (6.5–13.1)	10.7 (6.5–14.8)	0.367
	3 months	6.2 (4.1–8.2)	9.5 (2.2–16.9)	0.226
	6 months	3.5** (2.2–4.8)	9.1 (4.5–13.6)	0.005

P-value to indicate statistical significance from the starting value after 3 and 6 months of induction therapy in the group: * < 0.01, ** < 0.001.

**Figure 1:**
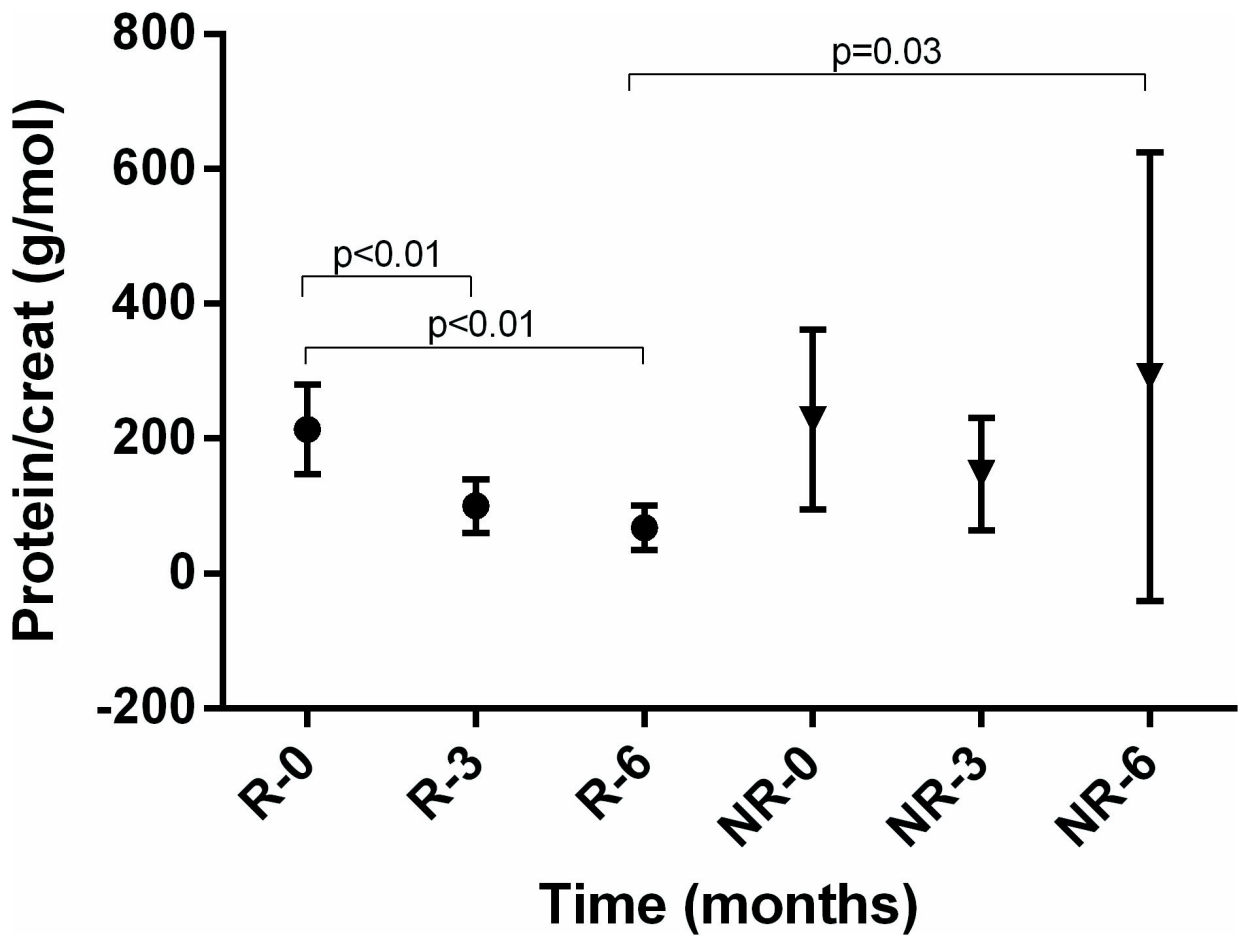
Mean urine protein to creatinine ratio in responders (R) and non-responders (NR) in time.

**Figure 2:**
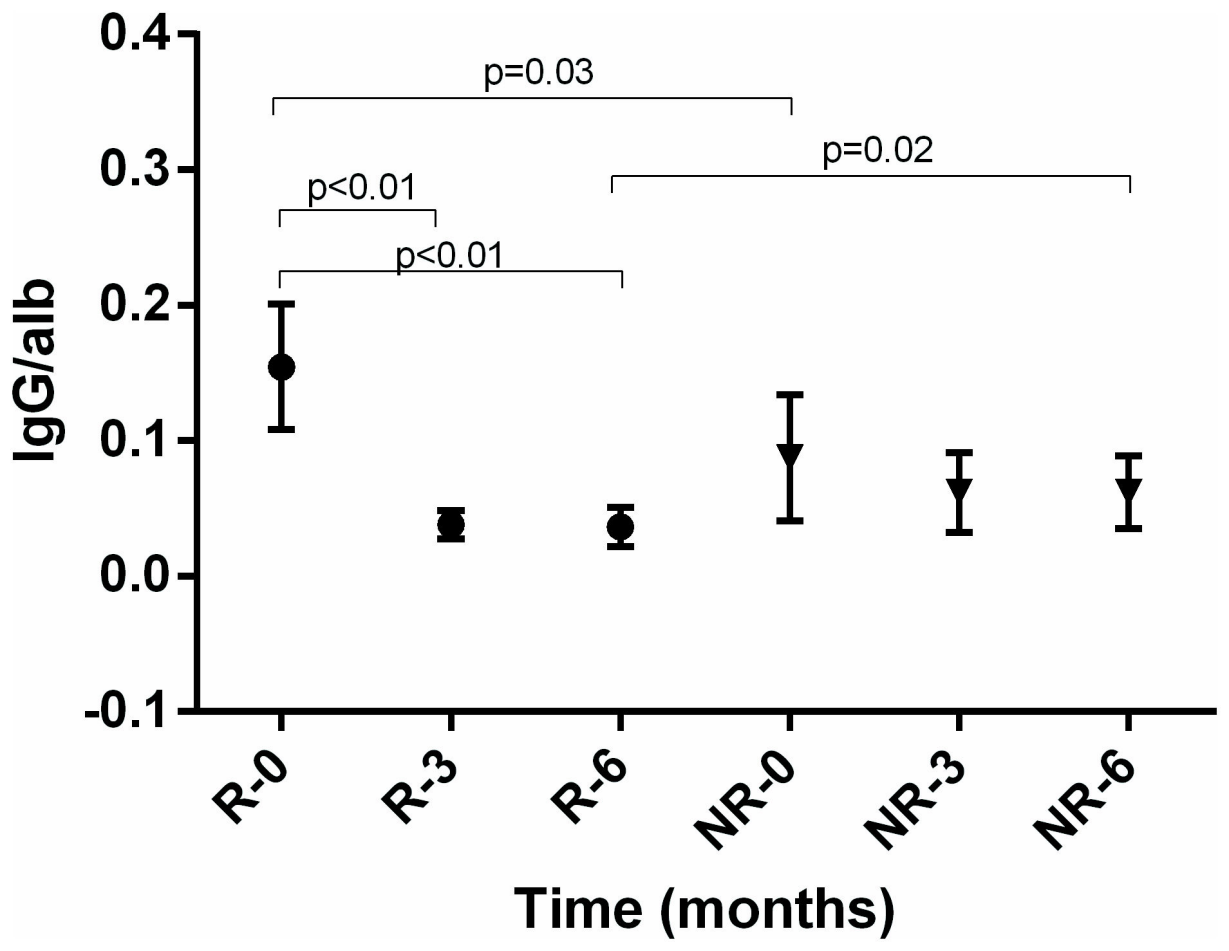
Mean urine IgG to albumin ratio in responders (R) and non-responders (NR) in time.

**Figure 3:**
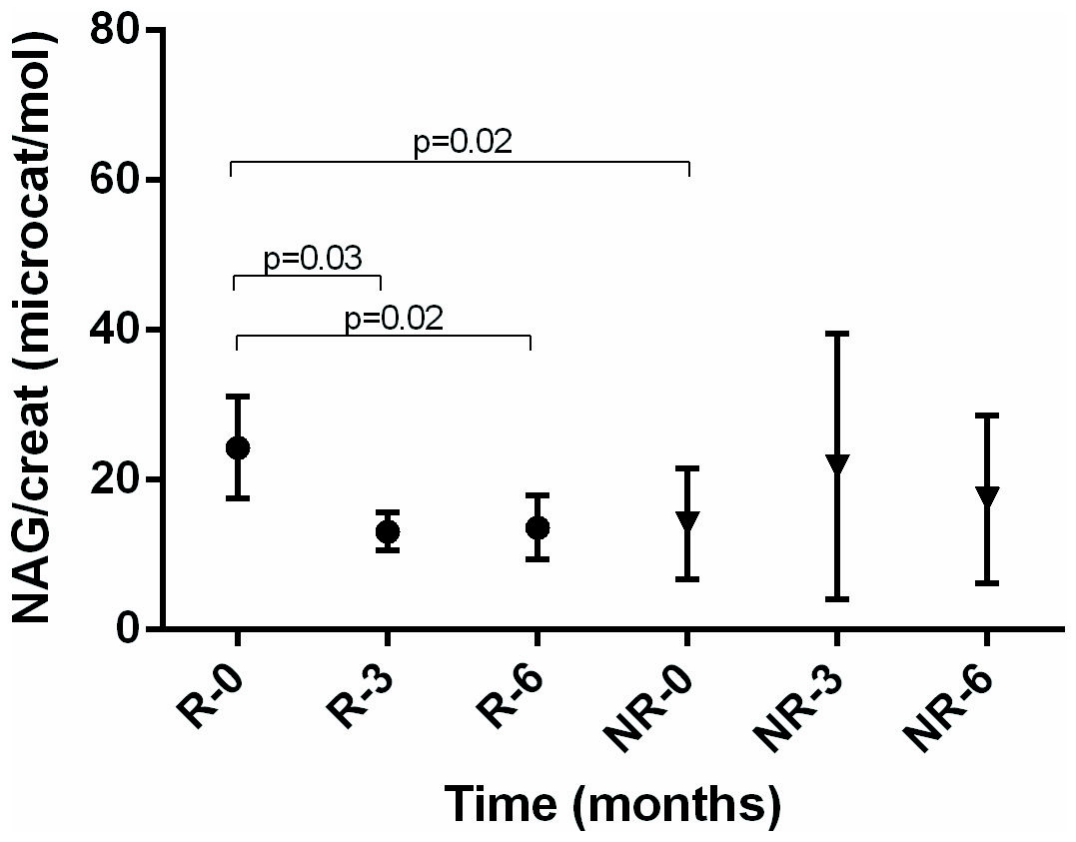
Mean of NAG to creatinine ratio in responders (R) and non-responders (NR) in time.

In Figures 1 to 3 we present the results of urinary ratios of protein to creatinine, IgG to albumin, and NAG to creatinine for the group of responders (R) and non-responders (NR) at the beginning of induction therapy and after 3 and 6 months of therapy.

### Predictive parameters for renal response at the time of diagnosis

**Figure 4:**
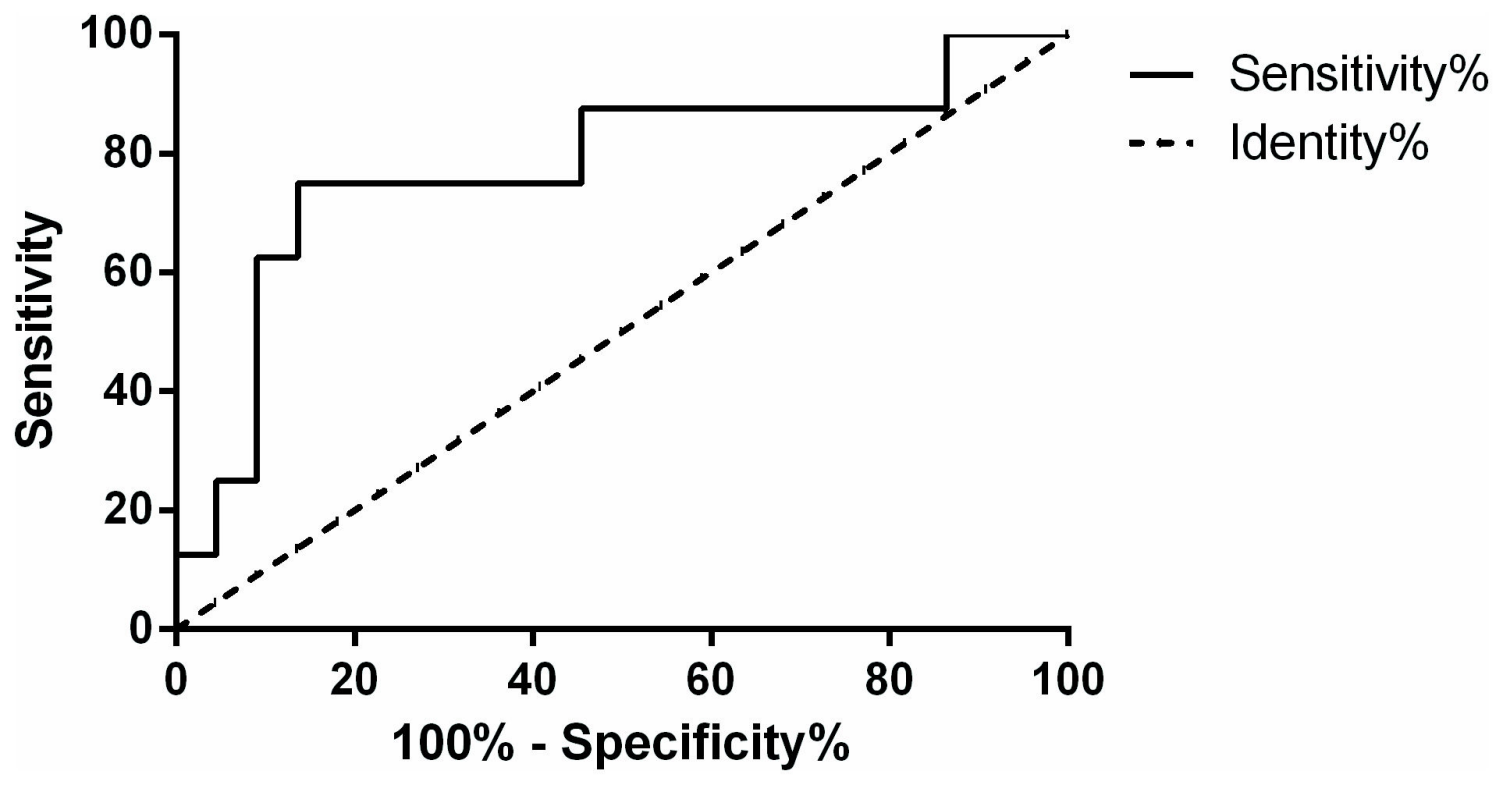
ROC curve with AUC for urine NAG to creatinine ratio at the time of diagnosis.

Area under the ROC curve for urine NAG to creatinine ratio was 0.78 (p = 0.021 and 95% CI 0.566 to 0.991) (Figure 4). Cutoff value for urine NAG to creatinine ratio at the time of diagnosis, with values above this limit suggestive of 5.5 times likelihood ratio for a good response to cytotoxic therapy, was 14.83 microcat/mol, with sensitivity 75% and specificity 86%.

**Figure 5:**
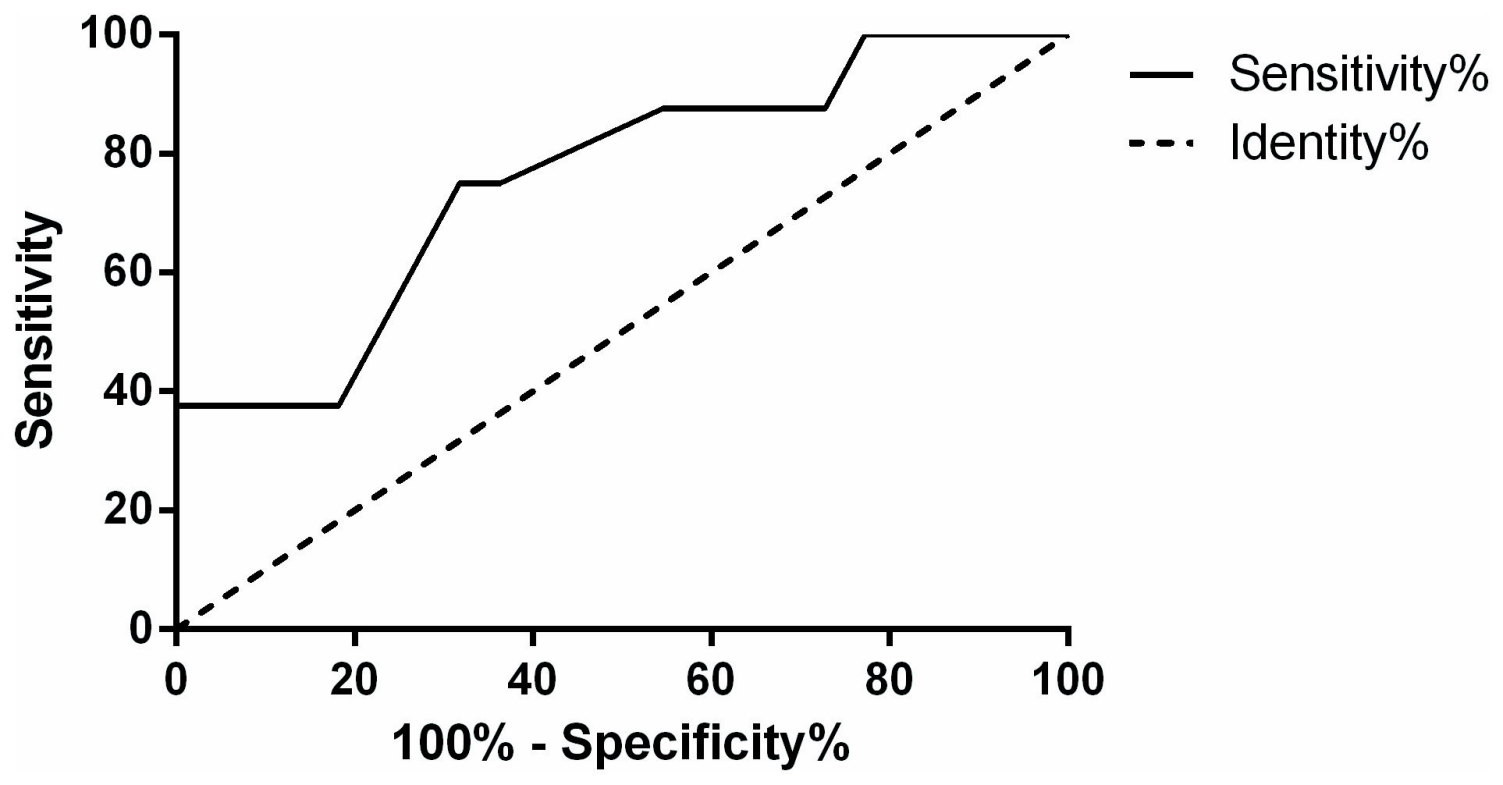
ROC curve with AUC for urine IgG to albumin ratio at the time of diagnosis.

Performing ROC for urine IgG to albumin ratio AUC at the time of diagnosis was 0.756 (standard error 0.102, p = 0.035 and 95% CI 0.556 to 0.955) (Figure 5). Cutoff value for urine IgG to albumin ratio at the time of diagnosis was 0.09, with values above this limit suggestive of a 2.3 times likelihood ratio of a good response to cytotoxic therapy, with sensitivity 75% and specificity 68%.

ROC for urine albumin to creatinine ratio at the time of diagnosis AUC was 0.636 (standard error 0.103, p = 0.260 and 95% CI 0.433 to 0.838), with cutoff value for urine albumin to creatinine ratio at the time of diagnosis 88.05 g/mol, with values above this limit suggestive of a 1.3 times likelihood ratio of a good response to cytotoxic therapy, with sensitivity 62% and specificity 54%. And ROC for urine protein to creatinine ratio at the time of diagnosis AUC was 0.558 (standard error 0.121, p = 0.646 and 95% CI 0.319 to 0.797), with cutoff value for urine protein to creatinine ratio at the time of diagnosis 159.6 g/mol, with values above this limit suggestive of a 1.1 times likelihood ratio of a good response to cytotoxic therapy, with sensitivity 50% and specificity 57%. Similar to proteinuria and albuminuria, all other parameters that were not different between responders and non-responders at the time of diagnosis (BVAS, eGFR, urine IgG to creatinine and urine α-1-microglobulin to creatinine ratio) were as such without predictive value for renal response at 6 months. 

### Predictive parameters for renal response after 3 months of induction therapy

There was a rise in eGFR after first 3 months of therapy in renal responders but no significant change in non-responders (Table 2). The relative change in eGFR at 3 months had a good predictive value for renal response at 6 months: ROC 0,84 (95% CI 0,67-0,95, p=0,0001). At the cut-off value of the rise in eGFR above 24% there was a 73% sensitivity and 88% specificity for a renal response at 6 months, positive likelihood ratio of 5.82. There was a significant fall in total proteinuria in responders after 3 months, but not in non-responders, which is mirrored also in similar changes of albuminuria (Figure 1, Table 2). In contrast to non-responders, there was also a significant fall in urine IgG to albumin and urine NAG to creatinine ratio in responders after 3 months (Figure 2 and 3). The AUC ROC values, optimal-cut-off values, sensitivity, specificity and likelihood ratios for the predictive capabilites of relative changes in urine protein to creatinine, urine IgG to albumin and urine NAG to creatinine ratios are shown in the Table 3. In 4 responders without significant improvement of renal function after 3 months of induction therapy, the reduction of urine NAG to creatinine ratio after 3 months announced a good renal response after 6 months of induction therapy.

**Table 3:** The ROC analysis of relative changes in various clinical parameters (measured at baseline and after 3 months of induction therapy) for predicting renal response at 6 months.

Parameter - relative change of	ROC AUC (95% CI)	p	Cut-off for prediction of response†	sensitivity	specificity	Likelihood ratio
Protein/creatinine (g/mol) (N=30)	0.67 (0.46-0.84)	0.3	N/A‡	N/A	N/A	N/A
Albumin/creatinine (g/mol) (N=24)	0.62 (0.4-0.81)	0.42	N/A‡	N/A	N/A	N/A
IgG/albumin (N=22)	0.73 (0.48-0.9)	0.14	N/A‡	N/A	N/A	N/A
α1-microglobulin/creatinine (g/mol) (N=23)	0.64 (0.42-0.83)	0.19	N/A‡	N/A	N/A	N/A
NAG/creatinine (microcat/mol) (N=23)	0.81 (0.6-0.94)	0.0057	<1.03	83%	80%	4.2
eGFR (ml/min/1.73 m^2^) (N=30)	0.84 (0.67-0.95)	0.0001	>1.24	73%	88%	5.82

^†^ the values for cut-off are calculated as ratios of the parameter at 3 months and baseline value of parameter

^‡^ N/A, for non-significant ROC AUC the cut-off values, sensitivity, specificity and likelihood ratios are not reported

### Maintenance therapy, renal remission and relaps rate

There was no difference in the maintenance therapy in responders and non-responders that were dialysis independent: 22 patients received azathioprine and MP, and 5 patients mycophenolate mofetil and MP. In 2 dialysis dependent non-responders with pulmonary involvement maintenance therapy with MP was continued, and in 1 dialysis dependent non-responder CyC and MP were discontinued after 3 months of therapy. 

Twenty six patients (86.7%) achieved renal remission after 12 month of therapy (all responders and 4 non-responders) (p = 0.002), and patient survival was 100%. In the period of 12 months, the cumulative dose of CyC was 1732.5 ± 530.2 mg/m^2^ in responders and 2212.3 ± 947.3 mg/m^2^ in non-responders (p = 0.16), and cumulative dose of MP 546.8 ± 377 mg/m^2^ in responders and 735.4 ± 423.7 mg/m^2^ in non-responders (p = 0.25). Four non-responders stayed hemodialysis-dependent after 1 year, and 2 responders became hemodialysis-dependent after 1 year (p = 0.03): one responder due to noncompliance and poorly controlled arterial hypertension and one due to concomitant renal diabetic disease without active renal vasculitis. 

The rate of renal relapses in the first 12 months was 6.6%: 1 responder relapsed 2 months after completion of induction therapy; one non-responder relapsed 3 months after completion of induction therapy (p = 0.48), both while receiving maintenance therapy.

## Discussion

In the present study we evaluated glomerular and tubular proteinuria and enzymuria as possible early indicators of good renal response to induction therapy with i.v. CyC pulse and methylprednisolone in patients with ANCA-associated glomerulonephritis. Our results indicate that as early as at the time of diagnosis, the urine NAG to creatinine ratio higher than 14.83 microcatales/mol and IgG to albumin ratio higher than 0.09 predicted a good treatment response to induction therapy. In contrast, conventional predictors such as initial proteinuria, albuminuria or eGFR did not have any early predictive value for therapeutic response. The remission of renal disease could be further anticipated as early as 3 months after introduction of induction therapy in patients with reduction of urine NAG to creatinine ratio below baseline value as well as in patients with at least 24% rise in eGFR. 

The immunosuppressive therapy is the cornerstone of treatment in patients with ANCA-associated vasculitis, but it is difficult to predict its therapeutic response and to individualize the therapy in order to reduce the side-effects. At the time of disease presentation, kidney biopsy can be helpful in predicting the response to therapy. Several studies demonstrated that the percentage of normal glomeruli in renal biopsies of patients with ANCA-associated glomerulonephritis are important prognostic parameters for estimating the preservation of kidney function in response to cytotoxic therapy [32,33]. In our study, there was no difference in pathohistological lesions in kidney biopsies between responders and non-responders at the time of disease presentation, and as such they did not predict the renal response. 

During the therapy and follow-up, BVAS is a helpful tool for monitoring the kidney response and overall disease activity and adjusting the treatment protocol if needed [27,28]. In our group of patients, the BVAS differentiated between responders and non-responders no sooner than after 6 months, while after 12 months of follow-up the score was significantly reduced in both groups of patients. 

Proteinuria and albuminuria are known as a major determinant of kidney disease progression [34]. In charge-selective proteinuria, the predominant finding is albuminuria or higly selective glomerular proteinuria. In case of size-selective proteinuria, the urine contains an increased amount of large proteins such as IgG or IgM and proteinuria is non-selective [14,35–38]. Non-selective proteinuria results in a higher degree of tubulointerstitial damage due to tubular cell activation with subsequent tubulointerstitial inflammation and development of tubulointerstitial fibrosis. This was already demonstrated in primary glomerulopathies as a correlation between high urinary IgM excretion to decreased GFR regardless of the degree of albuminuria, association of IgG excretion with the extent of tubulointerstitial damage, and NAG as a marker of tubular toxicity of proteinuria [23–26,39]. Higher NAG excretion was considered as a marker of treatment response to corticosteroid therapy in patients with primary glomerulopathies, such as membranous nephropathy, primary focal segmental glomerulosclerosis and minimal change disease [39]. In lupus nephritis undergoing oral prednisolone therapy, the reduction in urine NAG appeared later than the decline in proteinuria or improvement of eGFR and was in strong correlation with proteinuria [40,41]. Alpha-1-microglobulin is, similarly to NAG, considered as a marker of proximal tubular damage and has a negative predictive value for remission and progression in membranous nephropathy [25,42]. 

In ANCA-associated glomerulonephritis, a high level of urine IgM excretion was strongly associated with the development of end-stage kidney disease due to high percentage of glomerular crescent formation, the presence of necrotizing glomerular lesions and severe interstitial fibrosis [23]. On the other hand, in our patients with less selective proteinuria with higher urine IgG to albumin and higher urine NAG to creatinine ratio at the time of diagnosis, the renal recovery after induction therapy was better. A favorable renal response was seen as early as 3 months after induction therapy as a decrease in the NAG to creatinine ratio and rise in eGFR, and after 6 months as a decreased urinary secretion of total protein, albumin, IgG, NAG and α-1-microglobulin. It is possible that responders had a higher glomerular and tubular regenerative potential, without irreversible glomerular lesions and tubulointerstitial atrophy or fibrosis that cannot be ameliorated with immunosuppressive therapy. We suppose that in our patients with inadequate treatment response there might have been prolonged quiescent active inflammation and/or predominantly irreversible interstitial lesions with tubular atrophy and fibrosis without potential to reverse due to the chronic nature of the disease. 

The acknowledged and most efficient induction therapy is still the combination of CyC and methylprednisolone, but short- and long-term side-effects may limit its use. Therefore it would be beneficial to have a reliable marker of early treatment response to tailor the dose of cytotoxic drugs and the duration of induction treatment. That kind of approach would also limit the patients’ long-term exposure to toxic drugs, especially in case of relapsing disease like ANCA-associated vasculitis, where disease remission is safely maintained with less toxic drugs. Our approach enabled us excellent one-year patient survival (100%) and comparable renal survival and relapse rate with results of previous studies of patients with ANCA-associated glomerulonephritis [4–9,43–45]. On the basis of our results we can speculate that it might be possible to further improve the renal response with timely and individual adjustment of cytotoxic therapy on the basis of urinary biomarkers and eGFR at the time of the disease presentation and early in the course of the disease, and to lessen the toxic effects in the patients with inadequate treatment response with timely dose reduction or discontinuation when possible. 

In conclusion, in patients with ANCA-associated glomerulonephritis, urine IgG to albumin and urine NAG to creatinine ratio at the time of diagnosis, and urine NAG to creatinine ratio and rise in eGFR after 3 months of induction therapy proved to be better non-invasive predictors of good treatment response to induction therapy with i.v. pulse CyC and MP than conventional ones, such as proteinuria, albuminuria or renal histological changes. We suppose that on the basis of our results new possibilities for individualization of cytotoxic therapy in patients with ANCA-associated glomerulonephritis are likely. Still, our study is limited by its small size, therefore our promising results merit further investigation in larger prospective studies. 
